# Flatulent Dyspepsia

**Published:** 1890-03

**Authors:** Jose Nadal

**Affiliations:** Palamos


### FLATULENT DYSPEPSIA.

DR. JOSE NADAL, OF PALAMOS.

(TRANSLATED BY E. A. P.)

We shall treat of a loyal son of the village of Palamos, aged thirty-seven years, married, with four children, bilious-lymphatic temperament, educated, kind and amiable character. He does not know that during his childhood he had any serious illness, only the excessive abuse during his youth of purgatives, especially Brandreth's pills, which have destroyed the membrane of the intestinal tubes.

He was dedicated to the cork trade, which is the principal fountain of riches of this district. He had been going to Estremadura for the last ten years, staying there for ten months of each year, buying cork, renting cork plantations, collecting and classifying crops, and all preliminary work which is necessary for that industry. During these periods he has had at various times attacks of pernicious fevers, having to return to the bosom of his family in a truly alarming state. I remember at one time he was attended for a quarternary bilious fever that was controlled with Sulphate of Quinine in large doses besides many other medicines.

With all these antecedents, it can be well understood how this intestinal canal and its adjoining glands had suffered. Various attacks of periodical fevers cannot be suffered with impunity that are treated by powerful heroic medicines that must of necessity imprint a mark more or less profound in their passage through the organism.

He had called in more than twenty doctors. One diagnosed his disease dyspepsia, others lithiasis urica, others disease of the pancreas, others of the spleen, and each one according to his belief.

He had no need of my service until the 30th of April last—that on the occasion of visiting one of his sons. I found him just arrived from Estremadura, and after having related to me what he suffered proposed, if it was my pleasure, to submit himself to homœopathic treatment, to which I acceded with pleasure.

His symptoms are as follow: Good exterior aspect, healthy color, normal pulse, tickling sensation in throat, producing cough without any expectoration, good appetite, no thirst, and a saburral white coating on tongue. He was not free from decubitus, as sleep could only be induced lying on the right side. In the morning he was rather better excepting the weariness that remained after having passed a bad night, which he always did. His sufferings commencing between five and six in the afternoon (six hours after dinner), consisted of an intense pain that began around the navel, extending all over the abdomen, great swelling of the same radiating finally to all the lumbar region on both sides, obliging him to bend the trunk backward. These sufferings lasted nearly two hours, but by his account the greatest suffering began at one A. M. (five hours after supper), presenting the same symptoms as in the afternoon, but much more intense; the cruel suffering lasted an hour or more, falling asleep after he was relieved by vomiting his supper. With these volumes of symptoms I believed I was treating a case of flatulent dyspepsia. I was not assured whether there were renal complications, nevertheless I was inclined to so believe, taking into account that gravel had been passed several times at Estremadura. Considering one medicament alone could not cover this volume of symptoms, I prescribed Nux-v.[5] an hour before dinner and an hour before supper, and Lyc.[12] two hours after dinner and two hours after supper. The effect was such as could be expected from these two polychrests; the vomiting ceased from the first day and the pain, if there was any, was only a faint shadow of what it was. He passed four days and four nights in this satisfactory state until one day that he spent with his family on the shore near this village. He changed his diet; then his two medicines failed to produce a good effect. The pain returned in another form, accompanied with great swelling of the epigastric region. Carbo-vegetabilis dispelled rapidly these symptoms. I was obliged to suspend it to cure an anginal catarrh that came between, but soon was conquered by Belladonna.

After the angina more of the forementioned remedies produced good effects. Something had been done, as the vomiting had not reappeared, and the lumbar pain had all disappeared, but at the same hour as at first a pain in the sigmoid flexure of the colon with inflation from flatus as in the case of a hysterical woman. The patient seemed to feel a sore in this part of the intestines, and that the pain was produced by the friction of the excrements passing this spot. It was approaching the time for his return to Estremadura; he was in despair from what he suffered, and I no less, seeing that my desires were confounded by the inefficacy of my medicines. After all, considering that as a business man he might have suffered some reverses, and that this might help the cause in producing his illness, I proposed Ignatia amara[5]; the effect was magical; the pain appeared at six in the afternoon; he took the medicine, and on going down-stairs it had disappeared never to return. He remained here three days more without feeling the slightest pain, after which started for Estremadura where he continues in this satisfactory condition.

From *El Consultor Homœopatica*, Barcelona, September, 1889.

